# The effect of a probiotic on gastrointestinal symptoms due to menstruation in healthy adult women on oral contraceptives: randomized, double-blind, placebo-controlled trial protocol

**DOI:** 10.1186/s13063-022-06410-w

**Published:** 2022-06-10

**Authors:** Taylor C. Judkins, Marie-Laure Oula, Shireen Madani Sims, Bobbi Langkamp-Henken

**Affiliations:** 1 Food Science and Human Nutrition Department, University of Florida, 572 Newell Dr, Gainesville, FL 32611 USA; 2 Lallemand Health Solutions, 6100 Royalmount avenue, Montreal, QC H4P 2R2 Canada; 3 Univeristy of Florida College of Medicine, PO Box 100294, Gainesville, FL 32610 USA

**Keywords:** Women’s health, Gastrointestinal, Menstruation, Microbiome, Probiotic, *Bifidobacterium*

## Abstract

**Introduction:**

For many women, uncomfortable and stressful symptoms accompany the menstrual cycle each month, sometimes in a debilitating manner. Previous studies have reported that gastrointestinal symptoms in healthy women significantly differ by the day of the menstrual cycle, but few studies have assessed interventions intended to minimize these symptoms. Probiotics supplements have been shown to attenuate gastrointestinal symptom severity as well as self-reported feelings of stress in various populations. This study evaluates the effect of a probiotic on abdominal pain and gastrointestinal symptoms in healthy women who take an oral contraceptive, have regular menses, and typically experience these symptoms during menstruation with the primary aim being change in abdominal pain intensity related to the menstrual cycle with probiotic versus placebo supplementation.

**Methods and analysis:**

In this randomized, double-blind, placebo-controlled parallel study, participants will receive either a probiotic or placebo supplement. Participants will begin answering questionnaires approximately 7 days before the start of menstruation (i.e., active bleeding), and 3 days later, they will begin consuming the study supplement for 8 weeks. The questionnaires administered will collect data about abdominal pain severity (primary outcome) and duration related to the menstrual cycle, digestive health, dietary intake, stress, and digestion-associated quality-of-life. A subgroup of women will provide weekly vaginal swabs and stool samples to examine the effect of the probiotic supplement on microbiota composition and diversity for exploratory purposes. Two-sided tests using a linear model and a type I error rate of *α* = 0.05 will be employed to test all hypotheses. Continuous variables will be presented as means with standard errors and categorical variables, as counts or proportions.

**Ethics and dissemination:**

This study was reviewed and approved by the University of Florida Institutional Review Board 01. Written informed consent will be obtained from all participants prior to any study activities. Study findings will be disseminated at scientific conferences and publication in the trial registry or in a peer-reviewed journal. Any protocol amendments will be reported in the final manuscript of this study.

**Trial registration:**

ClinicalTrials.gov NCT04457401. Registered prospectively on 07 July 2020. The trial was completed in December of 2021.

**Protocol version:**

V4.0 (11-04-2020)

**Trial status:**

Currently recruiting. Recruitment began in November 2020 and extend until December 2021.

## Strengths and limitations

- This study is novel in that healthy women will be prospectively followed daily for an 8-week intervention period and will not be subject to recall bias.
- This study will be conducted in healthy individuals, defined as women who do not have a physician diagnosed gastrointestinal or gynecological disease. Previous studies have focused on women with various diseases or conditions such as endometriosis or irritable bowel syndrome, for example.
- A probiotic will be used to help mitigate gastrointestinal symptoms during the menstrual cycle.
- This study will recruit throughout the contiguous United States to ensure a more heterogeneous population.
- This study is limited in that only women who consume oral contraceptives qualify to participate. This may limit the generalizability of the present study to women who actively take combination oral contraceptives.
- The findings of this study may only have implications in women who do not have gastrointestinal and gynecological diseases. Future studies, however, may be conducted in other populations of women to present more broad findings.

## Introduction

Menstruation is a period of time that brings discomfort for many women. Women with various gastrointestinal or gynecological diseases or conditions may experience negative side effects from the menstrual cycle due to their diagnosis; however, many healthy women without these diseases or conditions may also experience uncomfortable symptoms. Healthy menstruating females experience several physiological symptoms on the first few days of menstruation such as abdominal pain, headaches, bloating, diarrhea, and constipation [1, 2] and have increased psychological concerns such as stress and anxiety [3]. While these symptoms are commonly recognized to affect a large portion of the female population, few interventions have been studied to assist in managing this discomfort. Some clinicians may direct their patients to take pain relieving medications without any thoughts on the consequences of chronic use [4]. When specifically consuming nonsteroidal anti-inflammatory drugs for menstrual related pain, the risk of adverse events such as nausea and indigestion is significantly increased compared to placebo consumption [5]. According to a recent study that prospectively characterized gastrointestinal (GI) symptoms throughout the menstrual cycle in women taking oral contraceptives, syndrome scores for abdominal pain, diarrhea, constipation, and indigestion (as measured by a modified daily version of the Gastrointestinal Symptom Rating Scale [6]) were all significantly different on the first day of menstruation than many other days during the menstrual cycle [7].

In addition to increased GI symptoms, self-reported stress also significantly increased on day 1 of menstruation and remained relatively high for the first few days of menstruation [7]. Stress has been correlated with GI symptoms through the gut-brain axis pathway [8]. Increased stress in the first days of menstruation is perhaps impacting GI function, and, in turn, GI symptoms related to menstruation may be contributing to overall stress. Severe abdominal cramping, such as dysmenorrhea, has also been associated with increased stress [9]. Dysmenorrhea is characterized as painful uterine spasms or severe abdominal cramps during menstruation [10]. Regardless of the order of occurrence, increased abdominal pain and stress on day 1 of menstruation may contribute to the observed increase in overall GI symptoms, increased stool frequency, and softer stool consistency observed in these women who were taking oral contraceptives (OC) [7].

Prostaglandins seem to be the major players in the induction of menstrual pain. Prostaglandin production and release still occurs in women on OC but to a lesser extent [11]. The GI tract may also respond to prostaglandin release during menstruation, which may explain changes in stool form and abdominal pain [12]. Prostaglandins may either increase or decrease intestinal motility, decrease gastric acid secretion, and modulate hepatic glycogen and glucose metabolism [12, 13]. The effects of prostaglandins may directly or indirectly contribute to the GI and emotional stress that is associated with menstruation. Regardless of the exact cause of GI symptoms and emotional stress, there is a need for an intervention that can help maintain physiological and psychological health throughout the menstrual cycle.

A proposed intervention to normalize GI function across the menstrual cycle is to supplement the diet with probiotics. Various probiotic supplements have been shown to effectively decrease diarrhea, constipation, stress, and intestinal permeability, as well as impact immunologic outcomes [14–16]. Probiotics also modulate stool form, as measured by the Bristol Stool Form Scale, to provide a more favorable consistency [17]. In particular, endogenous *Bifidobacterium* spp. vary throughout life in healthy humans and in disease states and have been associated with various beneficial health effects [18, 19]. Notably, *Bifidobacterium* strains are effective in decreasing stress, abdominal pain, and other gastrointestinal symptoms [14, 20–28]. Menstruation is generally associated with these types of symptoms [29–32], and specifically, stool form and self-reported stress are significantly different on the first day of menstruation compared with many other days throughout the menstrual cycle of healthy women on combination oral contraceptives [7]. Investigating the effects of a probiotic supplement in otherwise healthy women experiencing GI symptoms and stress during menstruation is warranted. Therefore, the primary aim of this study is to determine the effect of a probiotic on menstruation-related GI symptoms and self-reported stress throughout the menstrual cycle in healthy women on oral contraceptives.

### Objectives

The primary outcome of this clinical trial will assess the change in menstruation-related abdominal pain recorded over the first 3 days of menstruation while on the intervention compared with a similar time period during baseline between women on the probiotic versus placebo. Secondary outcomes will determine the effect of the probiotic on menstruation-related GI symptoms and function, self-reported stress, dietary intake, and food cravings. Exploratory analyses in a subgroup of participants will examine changes in the fecal and vaginal microbiome throughout the menstrual cycle.

## Methods and analysis

### Overall design

**Fig. 1:**
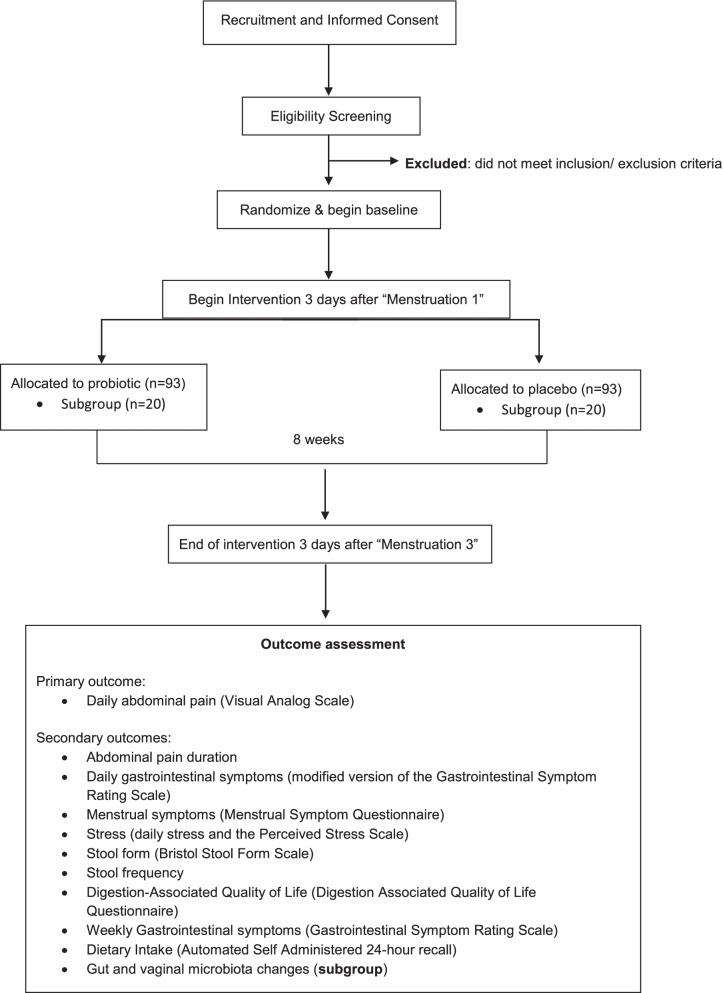
Schematic of the study design and flo

This 9-week randomized, double-blind, placebo-controlled clinical trial will be conducted online across the contiguous United States and at the University of Florida (i.e., exploratory subgroup providing biological samples), in accordance with the International Conference on Harmonizaiton-Good Clinical Practice (ICH-GCP) guidelines. This study observes a two-arm parallel design with a 1:1 allocation, including an exploratory subgroup providing fecal and vaginal swab samples. A diagram detailing the study flow is shown in Fig. 1. The protocol was prospectively registered on ClinicalTrials.gov; the database record (NCT04457401) will be updated to reflect any amendment to this protocol over the course of the study.

### Recruitment

Recruitment started in November 2020 and will continue over a period of 6 months. Healthy females aged 18 to 35 years who are taking OC will be recruited for this study, as OCs are expected to reduce the likelihood of unpredictable or unscheduled bleeding and to provide a more homogeneous population in regard to hormonal variability. The age range was determined to exclude women who may experience premature menopause [33]. Potential participants will be recruited by distributing printed flyers and handouts, sending emails, word of mouth, and by posting on social media. Participation will be open to anyone who meets the inclusion and exclusion criteria and has internet access. After informed consent is obtained from a trained study coordinator online using RedCap [34] and methods approved by the University of Florida’s Institutional Review Board-01, participants will self-screen based on the following criteria:

#### Inclusion criteria

- 18–35 years of age (inclusive);
- Regularly menstruating (i.e., approximately every 24 to 33 days);
- Typically have an average of at least mild abdominal pain (score of 3 or above on the GSRS screener) during the first 3 days of menstruation;
- Currently using a combination OC;
- Willing to discontinue the use of any probiotics, including supplements and foods labeled with “probiotic,” for the 2 weeks preceding the study and throughout, and to discontinue the use of fiber supplements during the study;
- Typically have one stool per day (subgroup).

#### Exclusion criteria

- Using a birth control implant, vaginal ring, shot, patch, or IUD;
- Lactating or known to be pregnant;
- Currently on treatment for a physician-diagnosed GI or gynecological disease or condition;
- Experiencing pain that is caused by a disorder of reproductive organs including endometriosis, adenomyosis, uterine fibroid, or a pelvic infection;
- Use of another investigational product within 3 months of screening;
- Antibiotics or probiotic supplements consumed within 1 month before screening;
- Allergy to milk, soy, or yeast.

Participants may withdraw from the study at any time. They are encouraged to contact a study coordinator to withdraw from the study. Some participants may discontinue study activities without notice, in which case they will be consider lost to follow up. The principal investigator may withdraw participants as well if they no longer meet the inclusion and exclusion criteria.

### Randomization, sequence concealment, and blinding

This is a double-blind study; the sponsor representatives involved in the study, the investigators, the participants, and any health care professional or site personnel involved in participant management or outcome assessment will remain blinded. Participants will be stratified on their typical abdominal pain score at the beginning of the study and randomized to either the probiotic or placebo supplement in blocks of a pre-specified size. This abdominal pain score will reflect the first 3 days of their typical menstrual cycle and will be on a scale of 3 (mild discomfort) to 7 (very severe discomfort). To maintain a balance in numbers of participants in the main and subgroup studies, randomization will be conducted separately for the main and subgroup studies. Participants will be stratified into two groups including those with mild abdominal pain (score of 3) and those with moderate to very severe abdominal pain (scores 4–7).

#### Sequence generation and allocation concealment:

The randomization sequence will be generated using a random-number generator and will be provided in opaque, sealed envelopes by an individual who will not have contact with the participants. Six unique four digit codes will be used for each supplement (6 different codes for the probiotic and six different codes for the placebo) in the event that an adverse reaction requires unblinding of one of the codes due to a participant reported adverse event. All supplement bottles will be identical and packaged the same way.

#### Implementation

After participants provide their informed consent and eligibility is confirmed, they will be assigned a study number based on the order that they consented. Participants will then be assigned to a week to begin the study based on when their next menstrual event occurs. Within each week, participants will be randomized by a co-investigator (TCJ) based on the order that they consented. Only the study number will be used during the study to identify participants, and all data will be permanently anonymized after study completion.

### Study conduct

The schedule of activities is presented in Table 1. After providing informed consent for the main group and subgroup, participants will self-report their height, weight, and typical fiber intake using a fruit, vegetable, and fiber screener (NutritionQuest) [35]. Participants will complete a demographics questionnaire where they will be asked to estimate the approximate date of their next menstrual event to determine their allocation date. All study days will be ± 3 days to account for variation in each participant’s menstrual cycle.

**Table 1:**
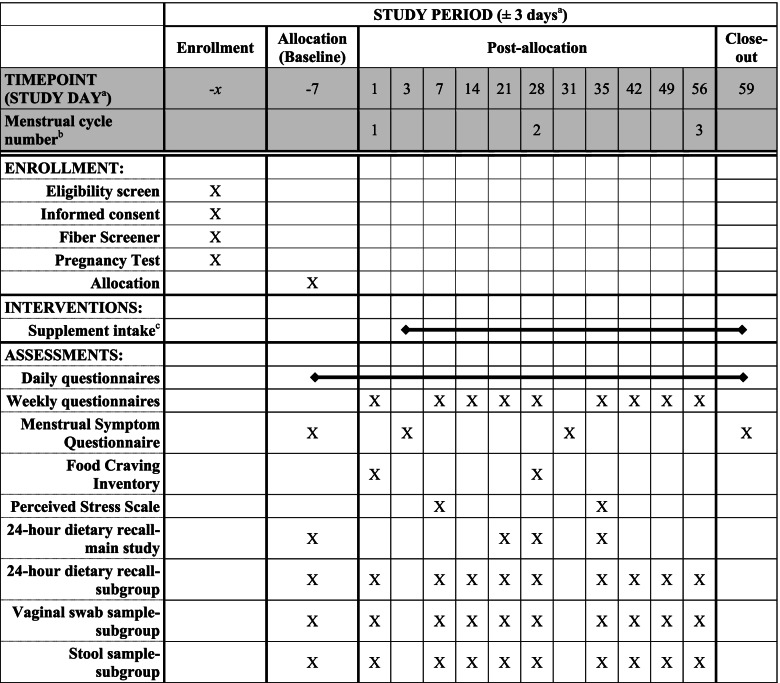
Schedule of activities

^a^Study days may vary due to variability in menstrual cycle length

^b^Each cycle and its assessments will be based on the first day of menstruation (first day of bleeding)

^c^Probiotic or Placebo

Following consent and approximately 1 week before menstruation, participants will be asked to complete a pregnancy test and will begin completing daily questionnaires. Briefly, daily questionnaires will inquire about GI symptoms, abdominal pain as measured by the visual analog scale [36–40], duration of abdominal pain, stress, stool form, stool frequency, adverse events, and medications taken, including the dose and administration schedule. Study days 3, 28, and 56 will be referred to as “menstruation 1,” “menstruation 2,” and “menstruation 3,” respectively. On the third day of their menstrual cycle (day 3/menstruation 1), they will begin consuming their study supplement (start of intervention). On the days participants experience GI symptoms related to the menstrual cycle, they will answer an additional questionnaire, which is a modified daily version of the GSRS to access GI symptoms. Approximately 7 days prior to menstruation 1 (study day − 7) and 3 days after each menstrual event (study days 3 and 31), participants will answer the Menstrual Symptom Questionnaire to assess symptoms leading up to and during menstruation. Seven days after each menstrual event (study days 7, 35, and 59), participants will be administered the Perceived Stress Scale to assess stress levels over the previous month [41]. Participants will also answer two validated weekly questionnaires including the GSRS and the Digestion-associated Quality of Life Questionnaire each week they are in the study. Each participant will also complete a 24-h dietary recall assessment at four points during the study (study days − 7, 21, 28, and 35) throughout the menstrual cycle. Finally, participants will also complete the Food Cravings Inventory (FCI) during the first and second menstrual event (study days 1 and 28). Participants will complete all questionnaires online using their assigned study number to maintain confidentiality. Questionnaires will be administered via Qualtrics Survey Software (Qualtrics Software Company) and data will be maintained on password-protected, encrypted computers.

While we will encourage participants to avoid pain relievers, consumption of over the counter pain relievers to assist with the pain associated with the menstrual cycle will be allowed for ethical reasons. We will ask participants to report any pain reliever consumption and instruct them to report their greatest abdominal pain intensity prior to consumption.

A subgroup of 20 participants per arm will also be asked to sign a separate informed consent form which will detail all of the main study tasks and will also include information on providing ten stool and vaginal swab samples during the study (study days − 7, 1, 7, 14, 21, 28, 35, 42, 49, and 56). These participants will be provided with stool and vaginal swab collection kits and receive instructions on how to self-collect these samples at home. Participants will be asked to return the samples to the study site. This subgroup will also complete additional 24-h dietary recalls (study days 1, 42, 49, and 56).

Participants will continue to answer daily questionnaires until the third day of their third menstrual cycle, at which point they will discontinue answering questionnaires and consuming the study supplement. At the conclusion of the study, blinding efficacy will be assessed via a questionnaire asking participants which supplement they thought they were consuming. Participants will be instructed to either return any unused capsules or text a picture of the remaining capsules to the study coordinator for compliance assessment.

### Intervention and compliance

Participants will be randomly allocated to either a probiotic or placebo supplement. The two supplements will look identical. The probiotic capsules will contain 3 × 10^9^ CFU of freeze-dried *Bifidobacteria* along with potato starch, magnesium stearate, and ascorbic acid as excipients. The placebo will contain only the excipients. Participants will keep the supplement at room temperature and will consume the intervention once a day with a meal for approximately 8 weeks. This is approximate to account for a few days’ variation in duration of individual menstrual cycles. Any adverse events that are believed to be due to the consumption of the probiotic will be assessed in daily questionnaires and reviewed by the study physician to assess unintended effects of the trial.

The study will be monitored monthly by the study sponsor through a data monitoring form. Study coordinators will monitor questionnaire completion daily to ensure participant compliance. If a participant does not complete a questionnaire, a study coordinator will contact them with a reminder. If the study coordinator notices that the participant did not consume their supplement, the participant will be contacted to remind them to consume their supplement. Compliance will be assessed by counting the number of study supplement capsules that were not consumed. Compliance is calculated by determining the number of capsules consumed divided by the number expected to have been taken to obtain the percentage consumed. In the daily questionnaire, participants will also be asked if they consumed the study supplement. In the event of a discrepancy between the information in the daily questionnaire and the amount of study supplement returned, compliance will be determined based on the product returned unless an explanation for loss of product has been provided. Participants found to have a compliance of < 80% will be considered noncompliant.

### Study outcomes

The primary outcome is the change in abdominal pain intensity related to the menstrual cycle at the beginning of the menstruation, calculated as the average of the first 3 days of “menstruation 2” when on the intervention minus the average of the first 3 days of “menstruation 1” prior to initiating the intervention. A daily visual analog score will be used to measure abdominal pain intensity between women on the probiotic versus the placebo interventions. We hypothesize that women consuming a probiotic will experience a greater decrease in abdominal pain compared to women on the placebo.

The secondary outcomes are defined as follows:

- To determine the effect of the probiotic on the number of days with abdominal pain (score above 0), assessed by the daily visual analog scale. We hypothesize that women who consume the probiotic will experience a decrease in the number of days with abdominal pain.
- To determine the effect of the probiotic on GI symptoms throughout the menstrual cycle, as measured by a daily modified version of the GSRS. We hypothesize that women who consume the probiotic will experience a decrease in the severity of GI symptoms compared to women who consume a placebo.
- To determine the effect of the probiotic on menstrual symptoms, as measured by the Menstrual Symptom Questionnaire. We hypothesize that women who consume the probiotic will experience decreased menstrual symptoms compared to women who consume a placebo.
- To determine the effect of the probiotic on stress, as measured by daily self-reported stress and a monthly Perceived Stress Scale. We hypothesize that women who consume the probiotic will experience decreased levels of stress compared to women who consume a placebo.
- To determine the effect of the probiotic on stool form and frequency throughout the menstrual cycle, as measured by the Bristol Stool Form Scale. We hypothesize that women who consume the probiotic will experience a more ideal consistency and pattern of bowel habits compared to women who consume a placebo.
- To determine the effect of the probiotic of digestion-associated quality of life, as measured by Digestion-Associated Quality of Life Questionnaire. We hypothesize that women who consume the probiotic will experience a better quality of life compared to women who consume the placebo.
- To determine the effect of the probiotic on dietary intake, as measured by the Automated Self-Administered 24-hour (ASA-24). We hypothesize that energy intake will not be different between the probiotic and placebo groups.
- To determine the effect of the probiotic on food cravings, a measured by the Food Craving Inventory (FCI). We hypothesize that women who consume the probiotic may experience fewer cravings than women on placebo.
- To explore the effect of the probiotic on the gut and vaginal microbiota in a subgroup of the study population. Recovery of the probiotic in the stool and vaginal samples will be assessed. Changes in the concentrations of the probiotic will be measured with specific DNA (or antibodies) in fecal and vaginal samples (e.g., qPCR, flow cytometry, or other methods). Effects of the probiotic interventions on overall microbiota composition (e.g., Illumina 16S rRNA sequencing) will also be measured. We hypothesize that women who consume the probiotic may show changes in microbiota composition compared to women on placebo.

### Data analysis

#### Sample size determination

As this topic of study is novel, it was assumed that a previously published study investigating the effect of ginger on pain relief in women with dysmenorrhea would provide an indication of the required sample size [38]. Abdominal pain scores as measured by the 100-point visual analog scale in the women receiving ginger was 58.01 ± 14.52. After 1 month on the ginger intervention, abdominal pain intensity decreased significantly to 43.49 ± 19.99. In the absence of data to estimate the standard deviation for expected change in pain score for the ginger, the standard deviation (19.99) for the final time point was used for the sample size calculation. A difference of 9 for the change in pain scores between intervention groups would provide an effect size of 0.5, which converts to approximately 69% of the control group to be below the average person in the experimental group [42]. We estimate that 79 participants per group will be required to find a 9-point difference in pain score between the probiotic and placebo with an alpha of 0.05 and 80% power. This sample size should allow for finding significant difference for change in pain scores between groups (i.e., the primary outcome). To account for a 15% drop-out rate, a total of 93 per arm for a total of 186 women will be invited to participate. Participants will be stratified on self-reported abdominal pain related to menstruation into groups of mild abdominal pain (score of 3) and moderate to very severe abdominal pain (score 4-7).

#### Statistical analysis

Intent-to-treat analyses will be performed for all outcomes with all eligible participants. A compliant per protocol analysis will be conducted on the primary outcome. Participants will be considered compliant if they consume > 80% of the interventional product during the 8 weeks of the study. Since there is only a single outcome measured, no imputation will be performed for missing variables. Statistical models that account for unbalanced data will be used if warranted, to account for participate drop out. Study results will be statistically analyzed by a statistical software programs (SAS and SigmaPlot). The mean delta (difference between pre and post-supplementation) will be tested by parametric or non-parametric *t* test depending on the normality of the data. Continuous variables will be presented as means with standard errors (SEM). Categorical variables will be analyzed as count or proportions. The significance of statistical tests will be determined using a type I error rate cut-off of 0.05. Categorical variable counts and proportions will be reported along with relevant standard errors.

#### Data monitoring

The study personnel will download and monitor data weekly to inspect for errors and compliance. The study personnel will also send a study monitoring report monthly to the study sponsor that includes information on the status of the trial, including adverse events. The study sponsor will have one data monitoring visit throughout the study.

#### Patients and public involvement

Patients and the public were not involved in the design of the study.

## Conclusion

To date, few studies have prospectively examined the effect of the menstrual cycle on the GI system in healthy women [7]. Menstrual cycles can be associated with many uncomfortable physical and emotional symptoms, as well as a significantly worse digestion-associated quality of life, which emphasizes the disturbance that the menstrual cycle can inflict on healthy women [43]. Approximately 80% of women experience pain due to the menstrual cycle and typically are only recommended to consume pain relievers and/or oral contraceptives, which do not come without side effects [5]. Probiotics supplements have been shown to attenuate GI symptom severity as well as feelings of stress, making this an appealing alternative to medications.

This study is novel in that it will prospectively examine GI and menstrual symptoms throughout the menstrual cycle. Each woman will have their own timeline during this study and will begin the trial based on when their menstrual cycle will begin. Previous literature of similar nature asked women to reflect over their past menstrual cycle, opening the door to recall bias [44]. This study will prospectively capture day to day physical and emotional symptoms and experiences. This study is also novel in that the focus is on healthy women. Often, healthy women and their menstrual symptoms are significantly overlooked because of societal attitudes towards menses. The menstrual cycle and its associated effects are viewed as normal because such a large population experiences these symptoms; however, they can significantly decrease quality of life even for women who are considered otherwise healthy.

This study will only include women who regularly take OC medication and who regularly menstruate every 24 to 33 days to allow investigators to better align daily data and menstrual events across participants. Validated weekly questionnaires must be administered within a certain timeframe, which this range will comply with. This cycle length also ensures that participants have “regular” menstrual cycles with enough time to accurately capture all of the data points. According to the International Federation of Gynecology and Obstetrics Menstrual Disorders Working Group, this range will also insure that women with irregular bleeding patterns are excluded from the study [45].

If this probiotic is effective in decreasing abdominal pain or stress associated with the menstrual cycle, the results of this trial could be useful to a large population of women. It is anticipated that this probiotic will attenuate GI symptoms and stress which is likely to increase quality of life during menstruation.

## Data Availability

The anonymized datasets used and/or analyzed during the study will be available from the corresponding author upon reasonable request and approval of the University of Florida.

## Abbreviations

ASA-24: Automated Self-Administered 24-hour Recall
BSFS: Bristol stool form scale
FCI: Food Cravings Inventory
GI: Gastrointestinal
GSRS: Gastrointestinal Symptom Rating Scale
OC: Oral contraceptives
